# Feasibility study of high-power electron linac for clinical X-ray ROAD-FLASH therapy system

**DOI:** 10.3389/fmede.2024.1382025

**Published:** 2024-06-16

**Authors:** Sergey V. Kutsaev, Ronald Agustsson, Salime Boucher, Paul Carriere, Nasr Ghoniem, Kenichi Kaneta, Maksim Kravchenko, Alan Li, Adam Moro, Sohun Patel, Ke Sheng

**Affiliations:** 1 RadiaBeam Technologies, LLC, Santa Monica, CA, United States; 2 Digital Materials Solutions, Inc., Carlsbad, CA, United States; 3 Department of Radiation Oncology, University of California, San Francisco, San Francisco, CA, United States

**Keywords:** X-ray radiotherapy, FLASH effect, electron linac, ultra-high dose rate, ROAD

## Abstract

**Introduction:**

This study examines how a practical source of X-ray radiation, capable of delivering unprecedented X-ray of 100 Gy/s at 1 m for X-ray FLASH radiotherapy can be designed.

**Methods:**

We proposed the design of a linac, capable of accelerating 18 MeV 8 mA electron beam with further conversion to bremsstrahlung X-rays. The design is based on L-band traveling wave accelerating structures with high power efficiency, operating in a short-burst/long-pulse regime that allows operating power supply in a regime, beyond its specifications.

**Results:**

This study demonstrates the feasibility of a high-power linac for a clinical X-ray FLASH therapy system, using detailed analysis and simulations. Despite ~500x higher output than a standard clinical linac, the design utilizes available accelerator components for maximal practicality.

**Discussion:**

Recent studies have demonstrated that the FLASH effect that allows to effectively kill tumor cells while sparing normal tissue occurs when large dose rates (≥40 Gy/s) are delivered in less than 1 s. Photons are very attractive since modest energies of several MeV are needed, which can be achieved with compact and cost-efficient accelerators. However, since the efficiency of electron-to-photon conversion is only a few percent, the required beam intensity must be an order of magnitude higher than that state-of-the-art accelerators can provide. The proposed ROAD-FLASH accelerator layout allows achieving both the FLASH dose rate and superior dose conformity, comparing to the similar projects. The current paper focuses on providing a technical roadmap for building an economical and practical linear accelerator for ROAD X-ray FLASH delivery.

## 1 Introduction

Radiation therapy (RT) in traditional cancer treatment aims to improve the effectiveness of treatment by selectively delivering a higher dose of radiation to tumors while minimizing damage to healthy tissues. This is achieved through precise targeting of radiation doses, using image guidance and radiation beams that deliver highly focused radiation. However, the effectiveness of treatment is still hindered by the harmful effects of radiation on healthy tissues due to entrance, exit and scatter doses. Successful radiotherapy relies on elevating the differential in cancerous and normal cell killing, which is termed therapeutic ratio. Therapeutic ratio can be increased by improving physical radiation dose conformity via technological evolutions, including Intensity-Modulated Radiotherapy (IMRT) (Webb, 1994; Sheng et al., 2006) Volumetric Modulated Arc Therapy (VMAT) (Nguyen et al., 2016), 4π non-coplanar radiation therapy (Dong et al., 2013a; Dong et al., 2013b), and proton therapy (Gu et al., 2018), but these technologies are limited by underlying physics and will plateau. Alternatively, improving radiation biological dose conformity has been explored as a means to improve the therapeutic ratio.

Conventionally, improvement of biological conformity has been achieved with tumor radiosensitizer or normal tissue radioprotectant. A new approach to enhancing the effectiveness of radiation therapy has recently emerged as the FLASH radiation effect (Vozenin et al., 2022). FLASH radiation therapy involves delivering an extremely high dose rates of therapeutic radiation at an accelerated rate within a fraction of a second. Research studies have demonstrated that when radiation is delivered at extremely high dose rates, healthy tissues appear to be consistently protected, while tumors are not (Favaudon et al., 2014). Although the specific conditions required to achieve this FLASH effect are not fully understood, current estimates suggest that radiation dose rates of >40 Gy/s delivered in under 1s can reduce normal tissue damage while maintaining tumor cell killing (Vozenin et al., 2022). The *in vitro* studies on 3D cell model have been recently successfully developed for investigation of a biological effect in healthy and cancer tissues (Durak-Kozica et al., 2023).

Regardless of the FLASH -therapy mechanism, the promising initial results warrant further investigation and human clinical trial studies. Nevertheless, there are significant technical challenges to achieving the orders of magnitude greater dose rate for FLASH in human patients (Schülte et al., 2023) The electron dose rates using existing linacs in the photon mode bypassing the target are high enough, but the achievable field sizes and energies are inadequate for most human applications and non-superficial tumors (Schuler et al., 2017). Certain proton systems can be modified to achieve the high dose rate but only in the dosimetrically inferior shoot-through mode that places the Bragg peaks behind the patient, due to the non-negligible time required to switch between energy layers in the proton scanning spot mode for volumetric coverage (Diffenderfer et al., 2020; Verhaegen et al., 2021). Even in the shoot-through FLASH mode of the existing proton system, only a fraction of the dose can be delivered at the FLASH dose rate due to small contributions from distant spots with lower dose-rates (Ramesh et al., 2022). Alternatively, passive scatter can be re-engaged to treat the tumor with FLASH dose rate using the spread-out Bragg peaks but this method is also dosimetrically inferior to state of the art scanning pencil beams (Zhang et al., 2021). These technical issues and the relatively limited availability of proton facility are barriers proton FLASH radiotherapy has to overcome.

An alternative tool for delivering FLASH could be an X-ray system. More than 90% of all radiotherapy is delivered with X-rays (Montay-Gruel et al., 2022), specifically, Bremsstrahlung X-rays produced by high energy electrons impinging on a target. They are the most versatile form of radiation therapy, and also the most cost effective. Unfortunately, the physical process for generating Bremsstrahlung X-rays is inefficient, therefore a high-power accelerator is needed (Kutsaev et al., 2021a) Furthermore, one would like to achieve the FLASH biological benefits with minimal compromise in physical dose conformity hinging on simultaneously achieving intensity modulation.

Despite the inevitable reduction in effective dose rate with intensity modulation and transmission through small apertures, a linac that can deliver 100 Gy in one second or faster is challenging but not impossible. Conventional 6 MV medical linacs produce a flattening filter-free dose rate of around 0.2 Gy/s at 1 m from the X-ray target—a 3 orders of magnitude too low—however they are on the low end of the spectrum of linac powers (Kutsaev et al., 2021b) A typical medical linac has a beam power on the order of 1 kW, while industrial accelerators for sterilization of food and medical products can achieve beam powers of several hundred kW (Kutsaev et al., 2021c).

Another factor that allows for improvement in dose rate is increasing the beam energy. The conversion efficiency from electron beam power to X-ray power scales approximately with E^3^, so a small increase in energy can make a big difference in X-ray intensity (Kutsaev et al., 2022). The increased X-ray energy also allows greater penetration. However, there are two major downsides to higher photon energies: larger lateral penumbra (Dawson et al., 1986; Ekstrand and Barnes, 1990) and greater neutron dose (Nath et al., 1984). That said, photon energies up to 20 MV are commonly used in RT. One could also consider reducing the distance from the source to the, however this can only be done to a certain point without sacrificing useability. Achieving good conformality requires that one or more collimators be placed between the beam source and the patient. That, along with pure physical limitations on fitting the equipment around the patient, tend to limit the source-to-surface distance (SSD) to 80 cm at the smallest.

**FIGURE 1:**
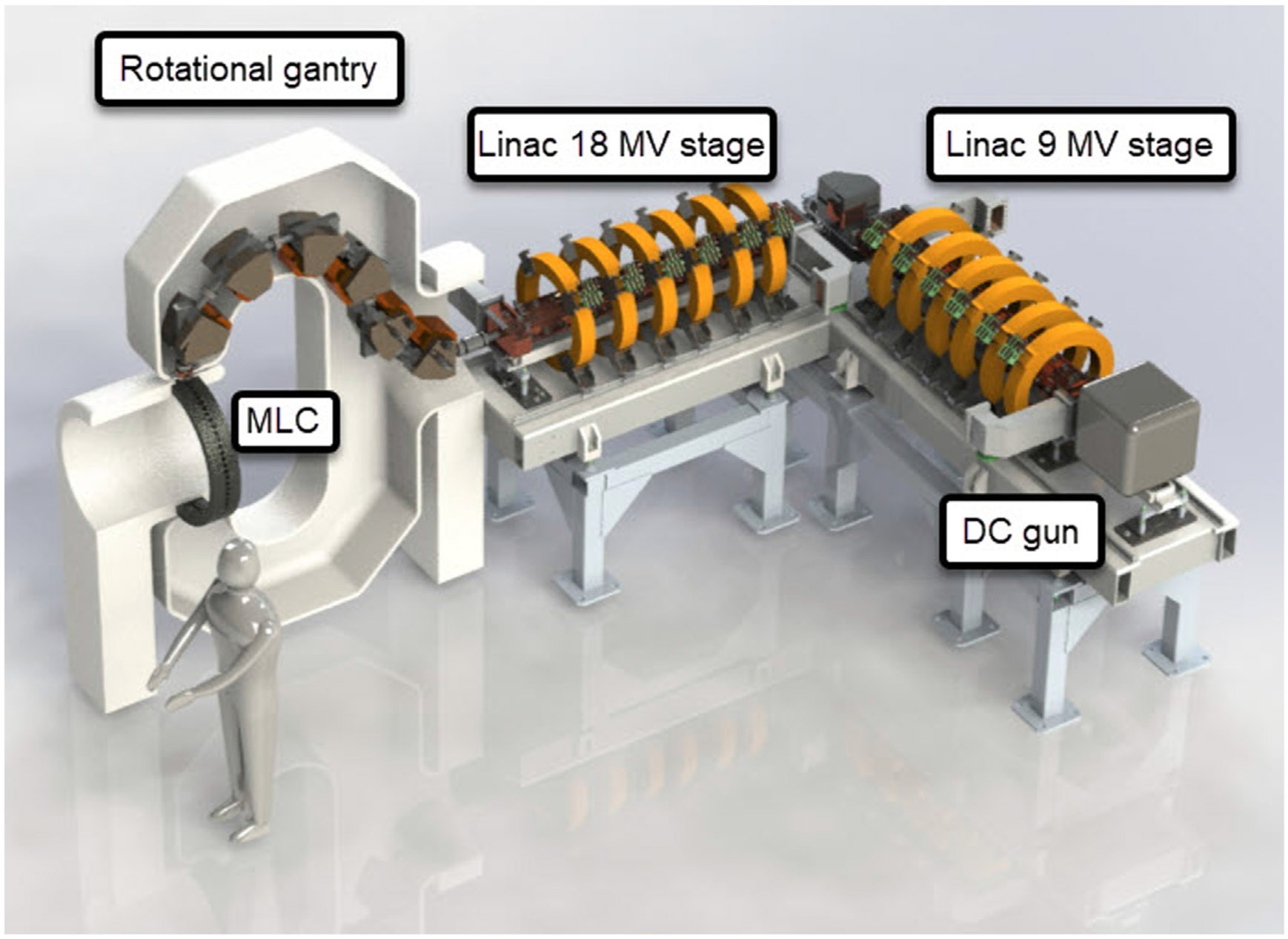
Rendering of the ROAD-FLASH system. The linac is triggered to produce the beam when the target is aligned with the MLC to produce VMAT-like treatment.

In response to these problems, an academic industrial partnership was formed to develop a solution for X-ray FLASH therapy that utilizes a single linac, which is based on proven accelerator technology, and employs an innovative and straightforward method for intensity modulation (Agustsson et al., 2021) Intensity modulation of the ultra-high dose rate X-ray would be achieved by a rotational direct aperture optimization system, known as ROAD, which involves a decoupled multi-leaf collimator (MLC) ring (Lyu et al., 2021). By rotating in opposite directions at a speed of 60 revolutions per minute (rpm), the linac and MLC rings deliver a total of 150 modulated beams within a single second. Each beam administers a dose of up to 0.7 Gy to the tumor. ROAD has the ability to achieve superior physical dose conformality, surpassing the dosimetry of current state-of-the-art volumetric-modulated arc therapy (VMAT) plans, while additionally benefiting from the FLASH effect. A model of the proposed ROAD-FLASH system is depicted in Figure 1.

Note that the high power linac would be significantly longer than what has been currently used in the clinic. Rotation of such a linac in the vertical plane would require a prohibitively tall vault. Instead, bending magnets are used to steer the beam while keeping the linac in the horizontal position. While the conceptual design of the ROAD-FLASH system has been generated, there are several key technical developments that require further investigation and development to demonstrate its feasibility. In particular, this paper focuses on linac design and optimization to enable the generation of the electron beam with sufficient beam quality for transport through the magnetic gantry and to achieve a <1.5 mm diameter focus on theX-ray target, and RF system design to power the linac, cost optimized for the atypical duty cycle (one treatment every 15 min).

## 2 Methods

### 2.1 Accelerator design

In order to estimate the accelerator parameters, such as beam energy, current, RF power etc., the following considerations were taken into account. The X-ray FLASH accelerator must be able to provide at least 100 Gy/s dose, collimated into the tumor, at 80 cm from the target. This corresponds to 256 Gy/s uncollimated dose at 1 m from the target, assuming modulation factor of 4, estimated based on intensity-modulated radiotherapy studies. The dose yield D is scaled linearly with the beam current I and cubically with the energy W as (Korenev, 2004):

$$D=k\cdot I\cdot W^{2.7-3.0}$$

Here, k is a yield factor that depends on the particular X-ray conversion target design. For example, at 9 MeV the dose conversion factor is 3 · 10^4^ cGy/min/mA (Author Anonymous, 1977), yielding the requirement for 50 mA beam (460 kW). At 12 MeV the conversion rate is 6.5 · 10^4^ cGy/min/mA, which corresponds to 23 mA beam (270 kW). Finally, at 18 MeV, the rate is 2.05 · 10^5^ cGy/min/mA, translating to 8 mA or 144 kW beam—a factor of 3 lower than for conventional 9 MeV energy.

100 kW-class industrial accelerators do exist and are in operation around the world (Jongen et al., 1993; Bryazgin et al., 2008; Yurov et al., 2024), but their dimensions are way beyond the hospital environment. On the other hand, these linacs are designed to work in continuous wave regime 24/7 in a factory setting with very little downtime. In the case of FLASH, the beam will only be on for a brief time, in the order of 1 s every 15 min, which significantly relaxes the requirements on, e.g., power supplies and cooling systems. As an example, that is very close to the requirements for the proposed FLASH delivery platform, we note that the CLIC drive beam injector parameters are 4 MeV, 4.2 A peak, 0.007 duty cycle, therefore 118 kW average beam power (Aicheler et al., 2012), operating in L-band frequency which is a compromise between the compactness, power availability, and power dissipation density. A commercially available 20 MW L-band klystron can supply enough RF power for the accelerator. Similar klystrons and modulators have also already been built and tested for the CLIC project (Marchesin et al., 2018), operating at 50 Hz with 150 μs pulses.

For example, in order to produce to beam duty factor of 2.5%, and peak current of 325 mA for the full duty cycle version of the FLASH system, the klystron with 167 μs pulses at 150 Hz can be used. For the available RF power, a linear accelerator can roughly produce a beam with the current and energy that scales as (Wangler, 2008):

$$P=\frac{W^{2}}{R\cdot L}+I\cdot W$$

For 18 MeV 325 mA, and R=40 MΩ/m for L-band structures (Adolphsen et al., 2014) this translates to 9.9 MW RF power in case of a 2-m-long linac. This power can be provided by E3736H klystron that operates at 1.3 GHz and yields 10 MW at 1.5% duty cycle (Canon, 2023). However, this klystron can operate at a duty cycle of 2.5% if limiting the irradiation time to 1-s operation, according to the communications with the vendor (Private Communications, 2024). The comparison of the requirements for the FLASH linac with a conventional RT linac is presented in Table 1.

**FIGURE 2:**
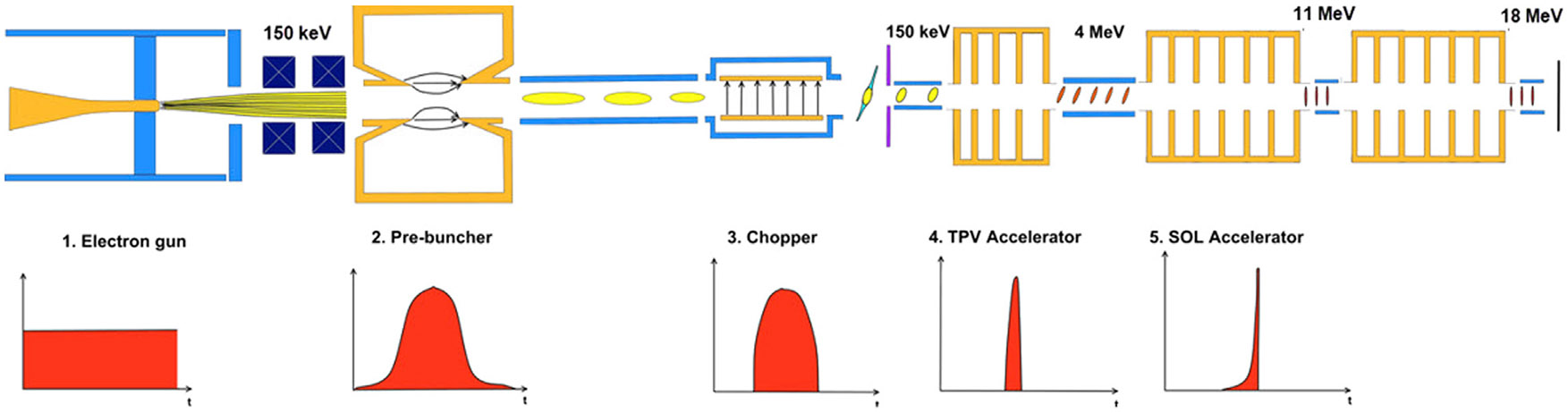
Schematic of the proposed ROAD accelerator (top) and the electron beam longitudinal profile development (bottom).

A conceptual design for the typical high-current linac is presented in Figure 2. While this design differs from those of standard medical linacs, the design is characteristic of those found in high-current industrial settings and scientific laboratories (Adolphsen et al., 2014; Kutsaev, 2021a) The thermionic electron gun with a DC Pierce geometry gun and gridded cathode can provide 1.5–3 A continuous beam, accelerated to 100–150 keV energies, so that losses in the injection system yield at least 25% of the initial current after the acceleration.

Next, it is necessary to reduce the current density by defocusing the electron beam after it emerges from the gun. Because of the high currents, it is necessary to maintain a beam radius under 1 cm to avoid second-order lens aberrations. This defocusing can be accomplished by a set of thin lenses (i.e., quadrupole magnets). After the electron gun, a number of bunching and focusing elements are configured to capture the DC beam into the RF accelerating buckets (Kutsaev, 2021a). These consist of a single-cell bunching cavity and a chopper, both operating at the harmonic RF frequency of 1.3 GHz. The bunched beam is then injected into a tapered-phase-velocity (TPV) accelerating section that compresses the bunches longitudinally, and accelerates them to ultra-relativistic velocities, so they can be further accelerated in the speed-of-light (SOL) accelerating sections before they reach the final energies. Solenoids need to surround the portion of the accelerator stretching from the buncher to the SOL to mitigate the repulsive space charge force and preventing beam-break-up (BBU) effects (Colombant and Lau, 1988).

In the following sections, we will demonstrate that the linac, designed, following the above-mentioned considerations can provide the beam, required for X-ray FLASH therapy and is feasible to build. This novel design that is much simpler than the conventional design, as it allows to eliminate pre-buncher and chopper elements, and integrate TPV section with a SOL one, therefore significantly reducing the number of components, their operational parameters and total accelerator footprint, which is essential for a clinical system. The accelerator design study includes RF cavity and beam dynamics simulations. We will also provide the results of the design optimization that allowed its simplification.

The primary task in the accelerator design is the selection of the accelerating structure. When dealing with MV-class accelerators with a few hundred mA currents, the standing wave (SW) structures are usually more compact and efficient than traveling wave (TW) structures. At the same time TW structures are preferred for high-current accelerators due to their stability and beam loading efficiency (Lapostolle and Septier, 1970) These structures are also more resistant to BBU effects (Meng et al., 2021; Gao et al., 2022).

**FIGURE 3:**
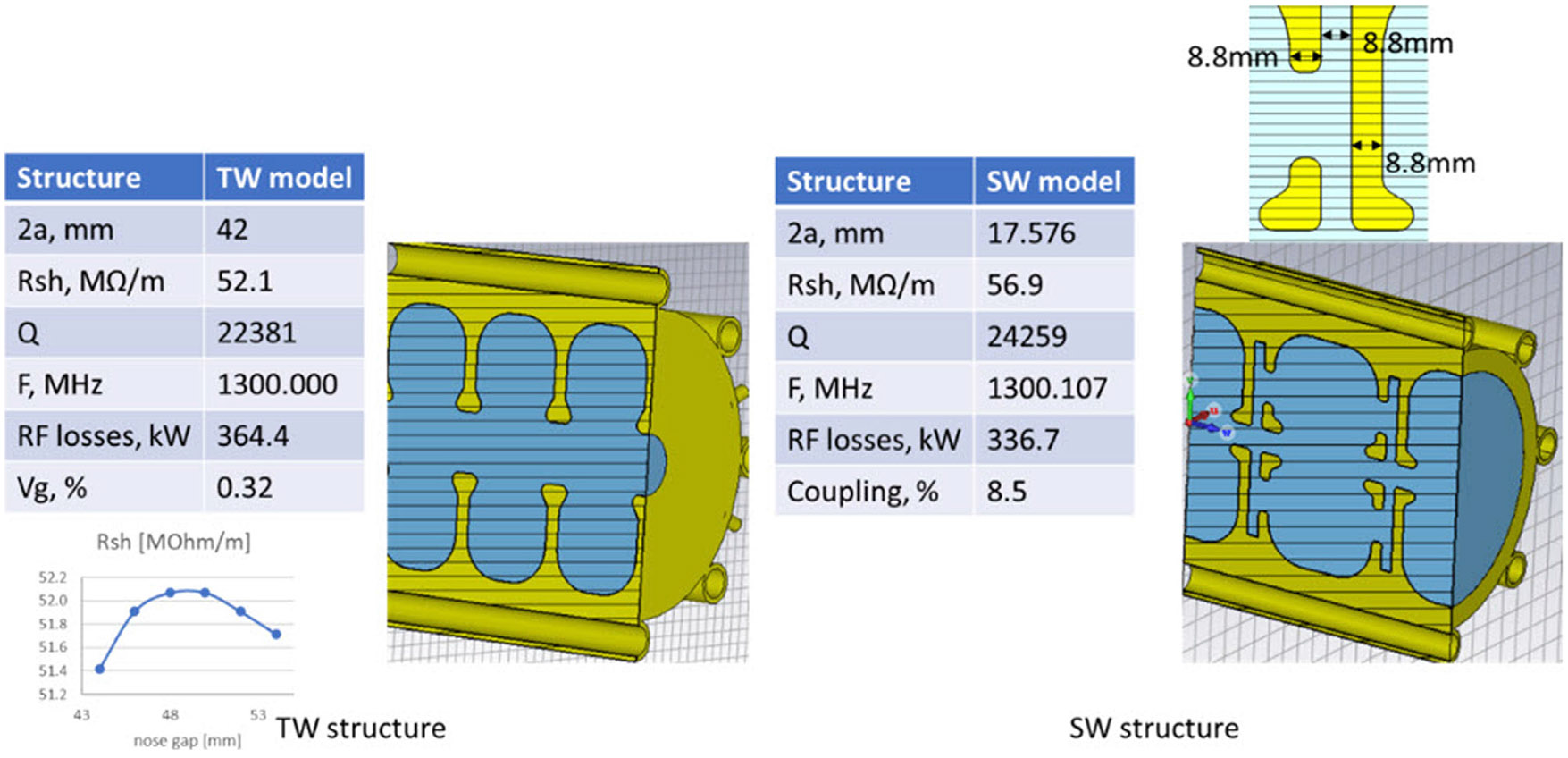
The cross-section of accelerating structures considered as candidates for a ROAD accelerator, and their RF parameters: TW disk-loaded waveguide (left), and SW on-axis coupled bi-periodic structure.

In our studies, we considered three types of accelerating structures, shown in Figure 3 (Kutsaev, 2021b): TW structure based on disk-loaded waveguide with constant Impedance (CI), i.e., uniform geometry to allow simplicity and constant gradient (CG) structure with a tapered aperture size to maintain uniform acceleration field distribution, and allow more efficient power utilization (Loew and Talman, 2008) The third considered structure was an SW on-axis standing wave accelerator, which was preferred to other structures due to simplicity and small transverse dimensions that allow the space for solenoid allocation (Sobenin and Zverev, 1999).

First, we estimated the total accelerator length for all three structures, using analytical expressions from (Kutsaev, 2021a), as it directly impacts its manufacturing cost and footprint constraints. The structure parameters used for the estimation were considered as follows. The SW structure is a scaled to L-band version of an S-band linac that we are currently building for the demonstration version of FLASH (Kutsaev et al., 2022). The TW structure has a geometry similar to a 40 MeV L-band linac for neutron production that is being built by RadiaBeam for Rensselaer Polytechnic Institute (Adolphsen et al., 2014), optimized for the highest shunt impedance ($R_{\text{sh}}$).

**FIGURE 4:**
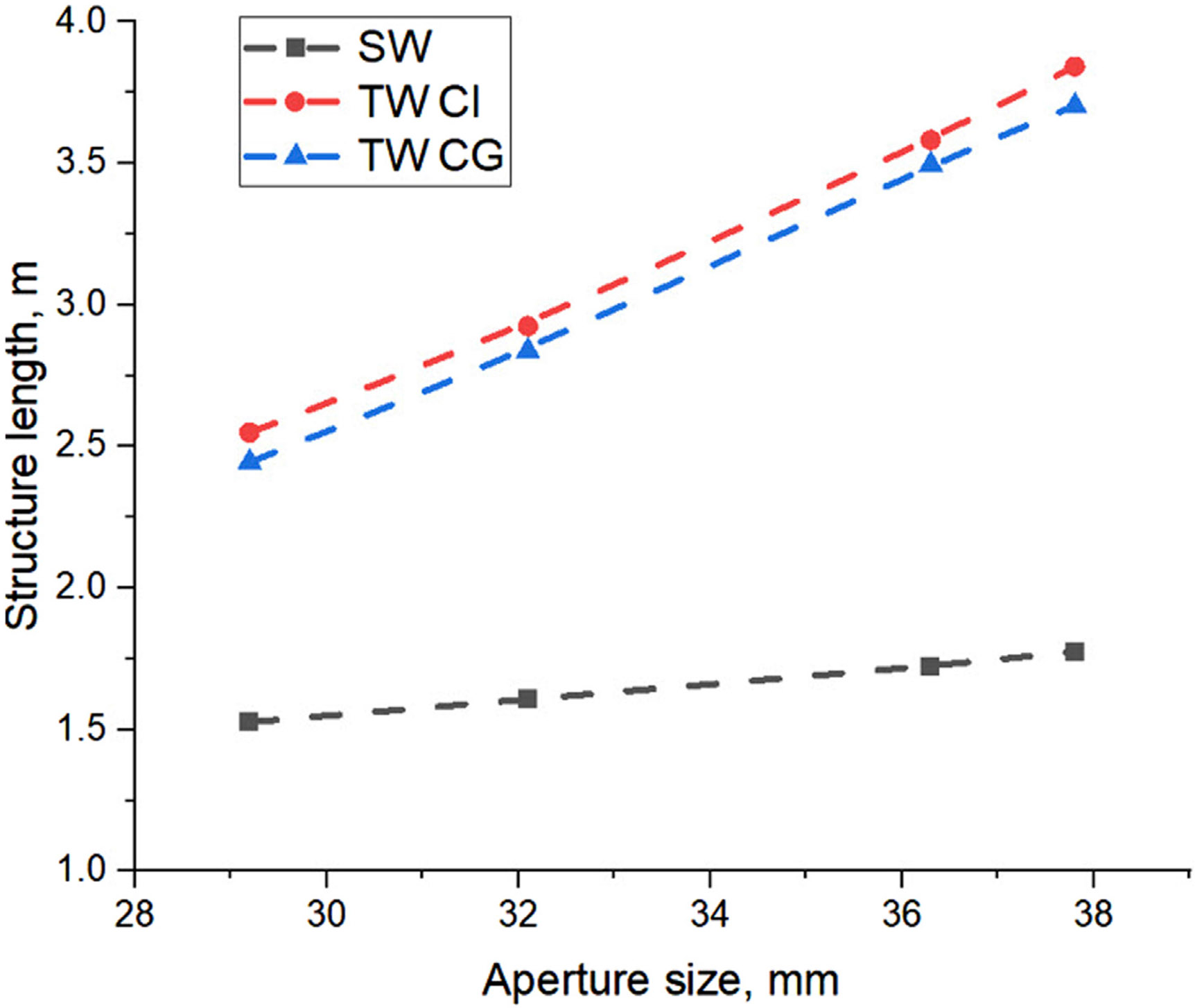
Comparison of total accelerating structure length, required to achieve 18 MeV in different types of the structures.

Analytical calculations, performed for all three structures and presented in Figure 4, show that the SW accelerator is shortest compared with TW accelerators. An important parameter here is the beam aperture diameter. Smaller apertures result in shorter structures due to the higher shunt impedance (Kutsaev et al., 2011), but the amplitude of the dipole mode, excited by the beam, that can cause BBU effect also scales a 1/a^4^ (Siy et al., 2022), and therefore, should be kept as large as possible. We also note that the manufacturing costs of the waveguide increase dramatically if its length exceeds 2 m due to the requirement for the large vacuum brazing ovens (Singh et al., 2012).

**FIGURE 5:**
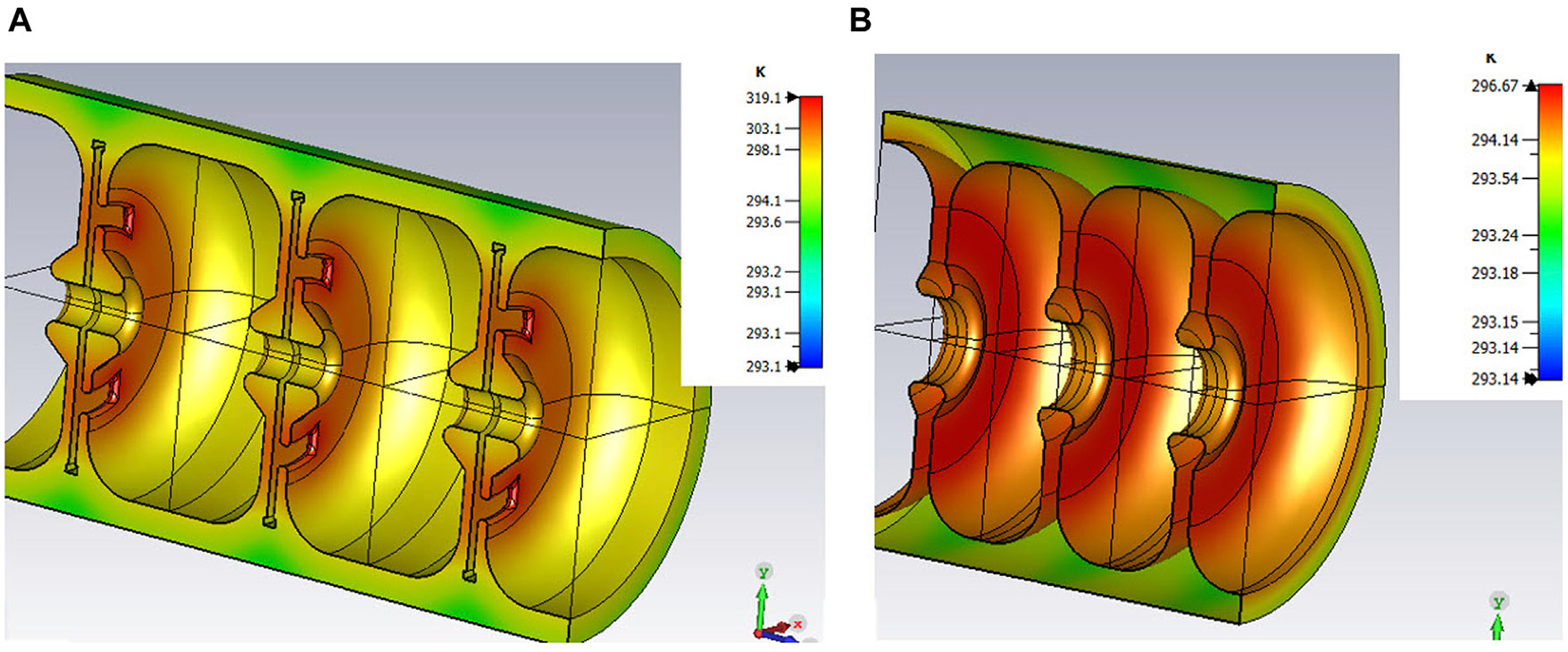
Temperature maps due to the heating of SW **(A)** and TW **(B)** structures.

Therefore, in order to decide, we need to consider other effects, such as frequency detuning due to the heating and beam loading, since the therapy will take only ~1 s, and there will be no opportunity for automatic frequency control (Cha et al., 2017; Kim et al., 2019) during the operation. In the case of a SW accelerating cell structure, 18 MeV can be achieved in 20 cells, with a wall-losses of 500 kW of power per cell with. The cell heating shown in Figure 5, and associated frequency shift due to the thermal expansion were calculated numerically in CST Microwave Studio and equals to −42 kHz. This frequency shift corresponds to the beam energy under-gain of 9.9%, according to the analytical model, described in work (Kutsaev, 2021a) In the case of a CG TW structure, the power losses are significantly lower due to smaller power density, and equal to 159 kW. As a result, the corresponding frequency deviation is −34 kHz and the energy under-gain is only 1.8%.

Due to significant frequency sensitivity and imminence of BBU, we have dismissed the SW option, even though it provides the most compact solution. When choosing between CI and CG TW options, we gave a preference to the CG structure. First of all, this structure is more efficient in terms of the linac length (Loew and Talman, 2008). Second, it provides uniform field distribution and therefore, precludes the beam lengthening effect due to the RF bucket enlargement, leading to smaller energy spread (Kutsaev, 2021a). Another advantage of the CG-type is that in such structures, the impedance of the accelerating and dipole modes changes from cell-to-cell unevenly, and therefore, the interaction between the beam and dipole mode is lower (Lapostolle and Septier, 1970), which increases the BBU threshold (Meng et al., 2021; Gao et al., 2022).

### 2.2 X-ray converter design

Accelerated electrons are used to generate X-rays for targeted radio biological testaments. As energetic electrons travel through a solid target material, they are decelerated by the action of the collective electric field of free electrons. The moving particle loses kinetic energy as photons (X-rays) are continuously emitted. When a target material intercepts the energetic electron beam, the beam’s energy is reduced as a result of its deceleration by electrons. Such energy loss can be determined from the stopping power of the material at the corresponding electron energy. The energy lost from the electron beam is converted to X-rays that may penetrate the target and end up deposited in human tissue for therapy or may be deposited locally in the material as is dissipated as volumetric heat within.

While the total energy lost from the beam can be accounted for accurately through knowledge of the stopping power, the exact split between local target heating and power of the radiation delivered to a patient requires detailed transport calculations. Dissipated electron beam energy in a target material can result in raising its temperature and the generation of corresponding thermal stresses. In extreme cases, the target material itself may melt or even vaporize if the deposited energy is not thermally managed properly. In this section we present a design of a spinning tungsten disk that can spread the heating from the accelerated beam over a larger area so as to avoid melting, evaporation, or disk fracture.

**FIGURE 6:**
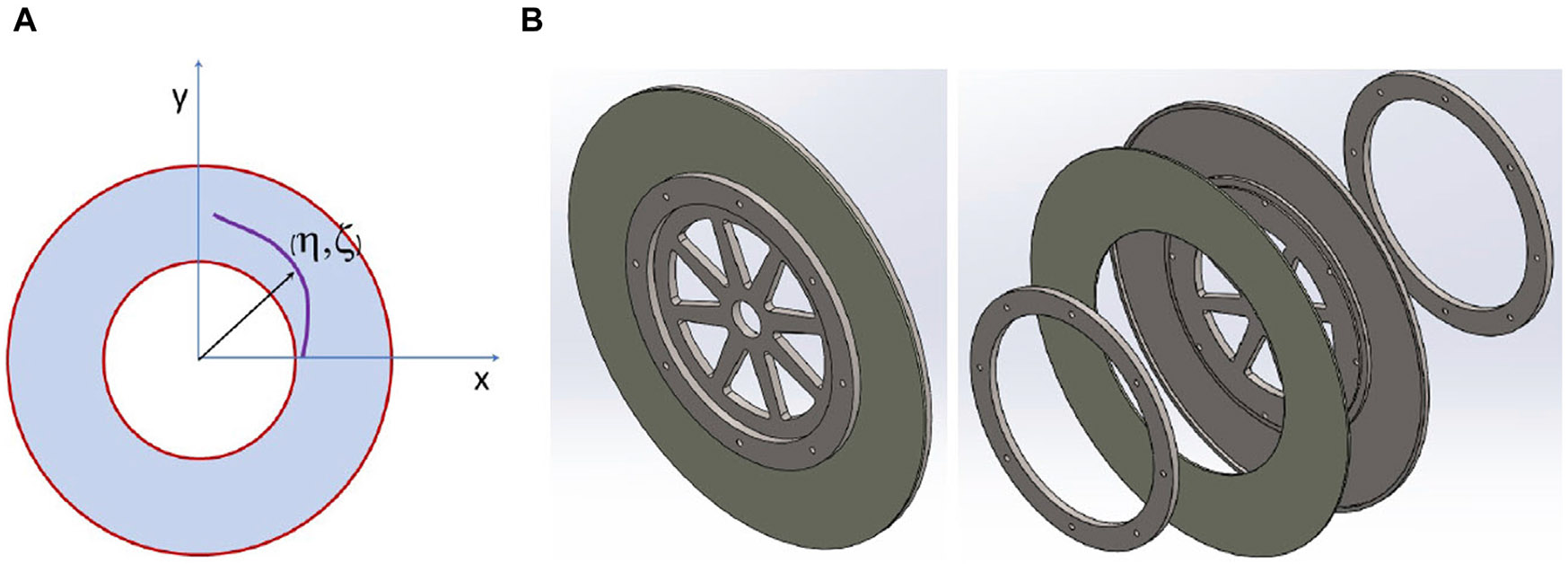
Spinning tungsten target geometry with coordinate systems **(A)** and its CAD assembly **(B)**.

Figure 6 shows the geometry of the disk and the Cartesian coordinates that describe the spiral center, (η, ζ), where η is the x-coordinate and ζ is the y-coordinate. To spread the electron beam heating over larger areas on the annular disk, one can spin the disk at a specified angular speed (ω) and at the same time move the beam or target either vertically or horizontally at a specified linear speed. This can create a spiral for the footprint of the electron beam center, and the spiral will grow as a function of time. The constraint here is the one needs the beam center to end up on the outer diameter circle within one second of operation.

One objective of the target design is its rotation speed. If the disk is rotated at a very low speed, many of the beam pulses would accumulate in a localized zone, which would result in more massive melting and large residual stresses that can lead to macroscopic cracks. On the other hand, if it is rotated extremely fast, the beam may strike in linear patterns as many pulses deposit their energy in a small spatial zone. Another objective is to design the disk with a small mass, so that the cost is low and the stresses generated by its rotation are minimized.

To estimate the parameters of the spinning target we use the following considerations. First, the energy dissipated by energetic electrons in materials can be determined if the stopping power is known. Using the tables of stopping power, ion range, and energy partitioning between nuclear and electronic losses. Provided by The National Institute of Standards and Technology (Author Anonymous, 2024), we estimate that at 18 MeV, the electron penetration depth is 4.1 g/cm^2^, which translates to 2.124 mm.

Because energy deposition from one single pulse occurs within 167 μs, and then the beam is turned off for 0.0065 s, we can determine whether one can assume that the deposited heat is uniformly spread over the spiral length as it covers the spinning disk. For example, as the thermal diffusivity of tungsten at room temperature is 2.8325 × 10^−5^ m^2^/s, and assuming a plate of thickness 2 mm, the time constant for heat diffusion is 31.5 ms. However, the thermal time constant for lateral (surface) heat diffusion in between pulses is determined by the distance between illuminated spots. The distance along the spiral direction is on average the beam diameter divided by the duty cycle (80 mm), and from one spiral turn to the adjacent is just the beam diameter (2 mm). Heat diffusion in between adjacent spirals will have a time constant of 15.8 ms. Although this analytical treatment can provide an initial approximate solution a more intensive study has been conducted and summarized below.

Although there are many solutions to the design of spinning disks of various dimensions and speeds, an optimum solution is desirable. Such optimal disk should satisfy several apparently conflicting requirements:

1. The disk must spin at the highest RPM possible to distribute beam heating during the pulse on-time and provide cooling through convective heat transfer.
2. The spinning speed must not be in resonance with the beam pulsing frequency.
3. The disk should have the lowest weight in order to reduce inertial forces.
4. The disk dimensions and size should be in a range that can be fabricated with conventional means.
5. The disk should be thick enough to avoid overheating during the 1s beam exposure without being so thick that it absorbs generated X-rays.
6. The disk should be durable so that the cost of replacement, disassembly, and assembly would be minimized.
7. The resulting beam heating pattern on the disk should be as uniform as possible to avoid steep temperature gradients and can be cooled down quickly in-between utilization.

## 3 Results

### 3.1 Accelerator

**FIGURE 7:**
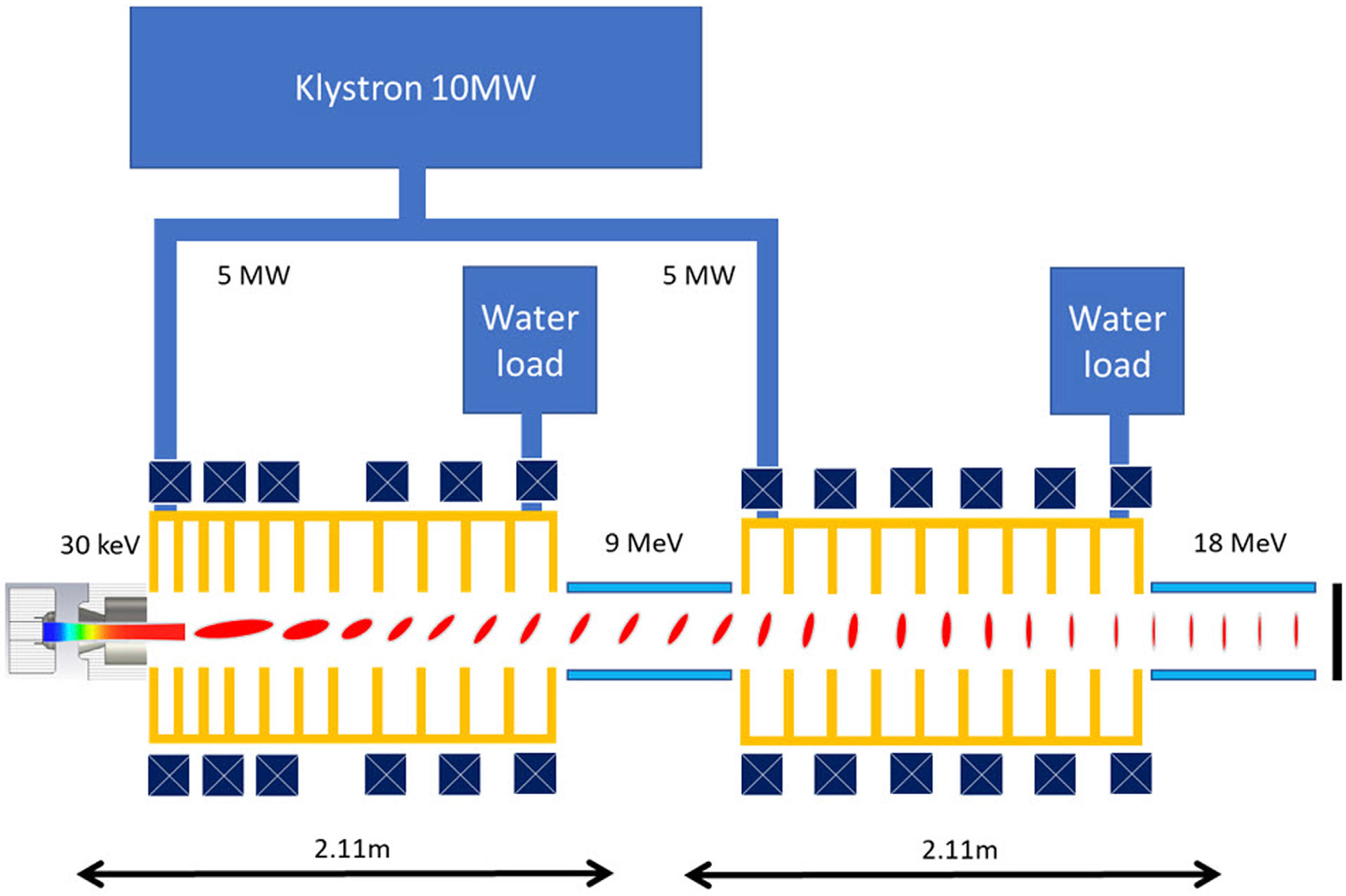
Optimized layout of ROAD FLASH accelerator. Two accelerated sections can be placed 90°–180° toward each other to provide more compactness.

The proposed improved layout of the accelerator is presented in Figure 7. It consists of 2 TW CG 2-m-long sections to facilitate the brazing. The first section also has the integrated bunching section. First, this decision provides a smaller footprint, compared to the conventional layout, presented in Figure 2, as it eliminates the pre-buncher, buncher and chopper elements. Second, it introduces further non-linearity into impedance distribution, further improving the resistance to BBU (Gao et al., 2022) This layout will, however, result in larger beam energy spread in the integrated bunchers is larger, since the DC beam will occupy the full RF bucket during the bunching. Then, the energetic particle losses in the bunching section due to the fact that only a fraction of the DC beam will be accepted for electron acceleration, causing excessive X-ray generation in this section that will need to be countered by additional shielding (Barish, 2014) On contrary, the chopped beam in a conventional layout is entirely injected into RF bucket (stable region of beam oscillations), so all losses occur in the chopper, where the particles have the energy of DC gun (up to 150 keV).

At the same time, the chopper eliminates a significant (50%–75%) portion of the beam, so high current Ampere-class DC guns that operate at >100 kV voltages are required. The guns rated higher that 30 kV require special certification in the United States, while the 100+ kV guns are very complex and require significant isolation (Zhou et al., 2014) Therefore, by making a decision to have the integrated buncher and accepting the associated drawbacks, it is possible to significantly reduce the requirements for the injection voltage, further simplifying the design, which is important for a clinical system.

**FIGURE 8:**
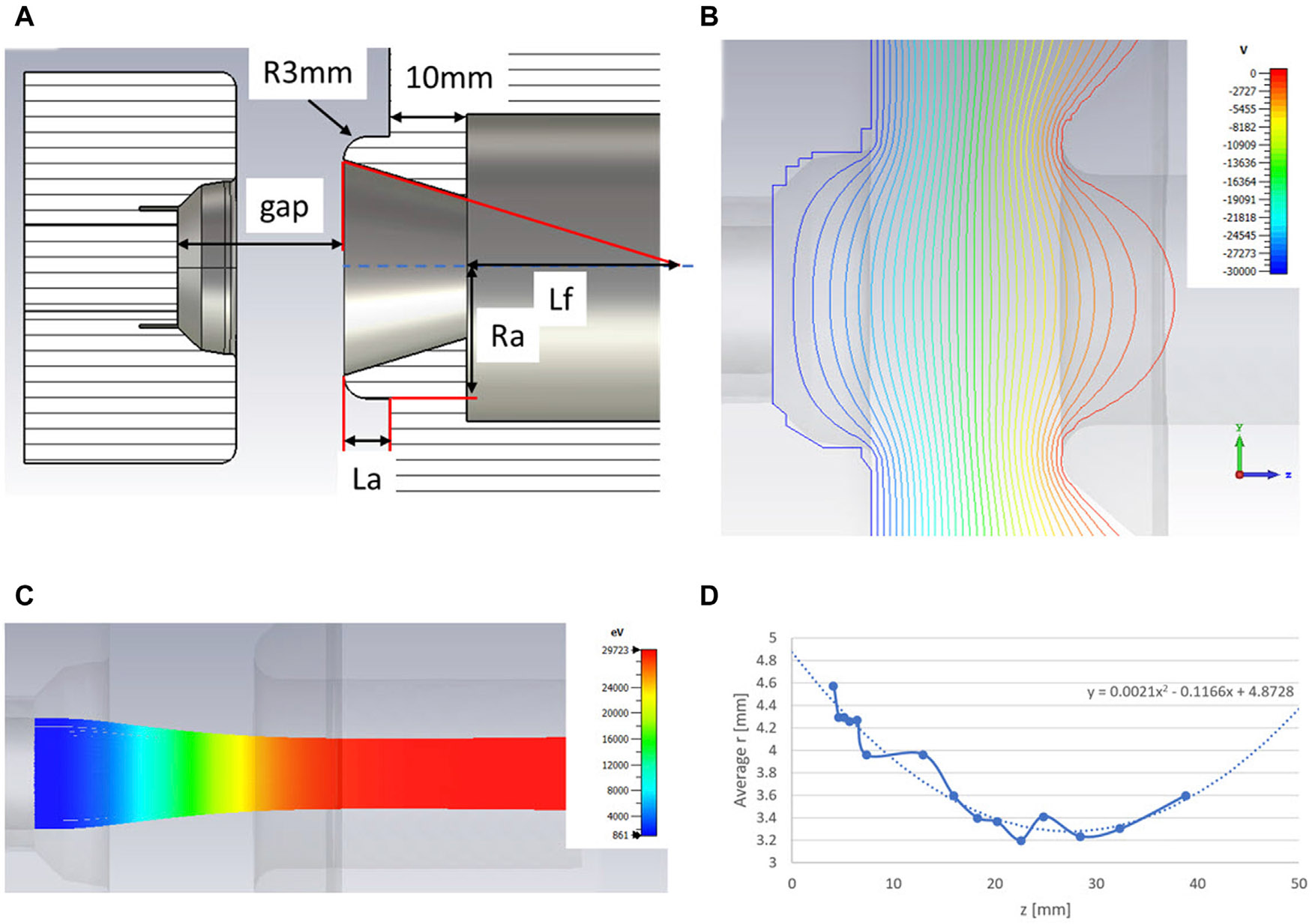
Design **(A)**, field distribution **(B)**, beam trajectories **(C)** and waist position **(D)** of the 30 kV DC gun.

For an injector, we will use a 30 kV triode electron gun, currently employed in the prototype linac for FLASH effect demonstration (Kutsaev et al., 2021c; 2022) This gun provides beam emission of up to 3 A, allowing very relaxed requirements for beam transmission through the whole linac. We optimized the anode geometry as shown in Figure 8 by adjusting the anode tapering dimensions. This design provides the focusing point (beam waist) in the middle of the first accelerating cell, which is the most preferable for beam dynamics (Zavadtsev et al., 2011; 2013).

We have also estimated the required solenoid field for BBU suppression, according to SLAC methodology (Chu, 1966). With no solenoid, the threshold current for 18 MeV, 1.3 GHz, 4-m long section with the effective beam aperture of 2.1 cm and the structure RF parameters, provided in Figure 3, is estimated to be 20 mA. The required solenoid field to increase this threshold to 325 mA is estimated to 0.8 T. In order to reduce this field to a more practical limit of 0.185 T, the beam aperture size must be increased to 3.5 cm (Smirnov, 2008).

In the following section, we provide the start-to-end simulation results of the linac that will demonstrate the feasibility of the proposed approach.

The design of a TW linac starts with the definition of accelerating cell parameters along the structure according to the following considerations. First, the aperture size must be as large as possible to increase group velocity, and therefore reduce dimensional sensitivities (Smirnov et al., 2023) Additionally, larger aperture allows higher beam transmission and reduce beam losses inside the linac. On the other hand, the accelerating field gradient, defined by the beam aperture, must be high enough to provide an overall length of less than 2.0 m and accelerate 325 mA beam in 9 MeV. Finally, the aperture must be reduced from cell-to-cell to compensate for the field drop due to the wall-losses and beam loading effect (i.e., the energy transfer from the RF field to the beam) (Lunin et al., 2011).

**FIGURE 9:**
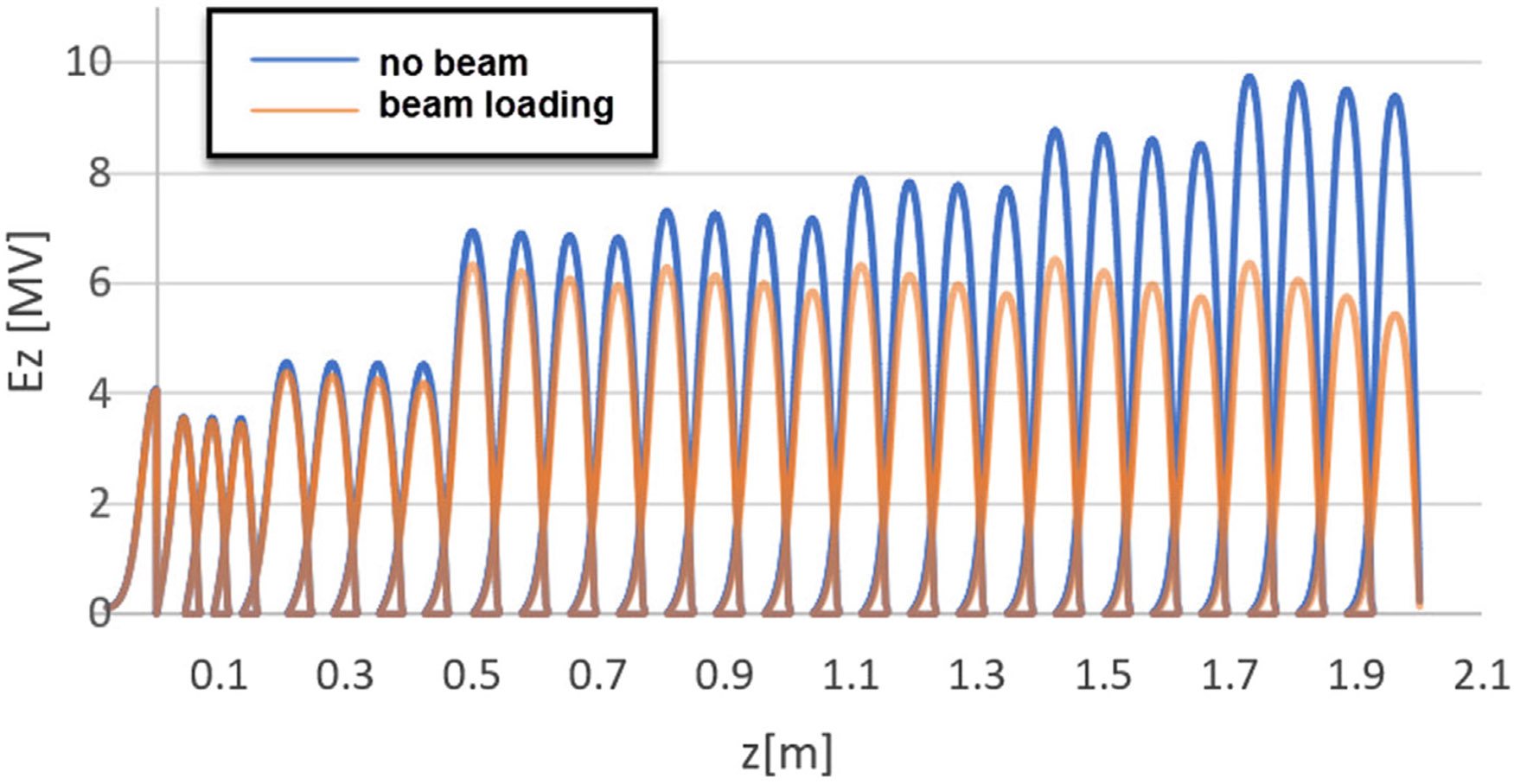
Accelerating field profile of the optimized TW CG structure, demonstrating constant accelerating gradient after considering beam loading effect (orange plot).

Next, the buncher was optimized in order to maximize beam transmission and minimize energy spread. The following parameters were considered during the optimization: phase velocities of the first 8 cells and field amplitudes in these cells. Two criteria were used for the optimization quality: beam transmission—should be maximized, and beam spectrum width—should be minimized. The approach for the buncher optimization is described in the works (Kutsaev, 2010; 2021a; Kutsaev et al., 2022) The best configurations of the bunchers and corresponding beam parameters are summarized in Table 2 and Figure 9. Next, we designed the second acceleration section to accelerate the beam from 9 MeV to 18 MeV. Again, the bore diameter was made as large as possible, and the total length was designed to be less than 2.0 m so that the electric acceleration field would be uniform. The second section doesn’t have a bunching section and consists only of SOL cells. The filling times of the first and second acceleration tubes are 744 ns and 739 ns, respectively, which are sufficiently small compared to the RF pulse width of 167 μs.

**FIGURE 10:**
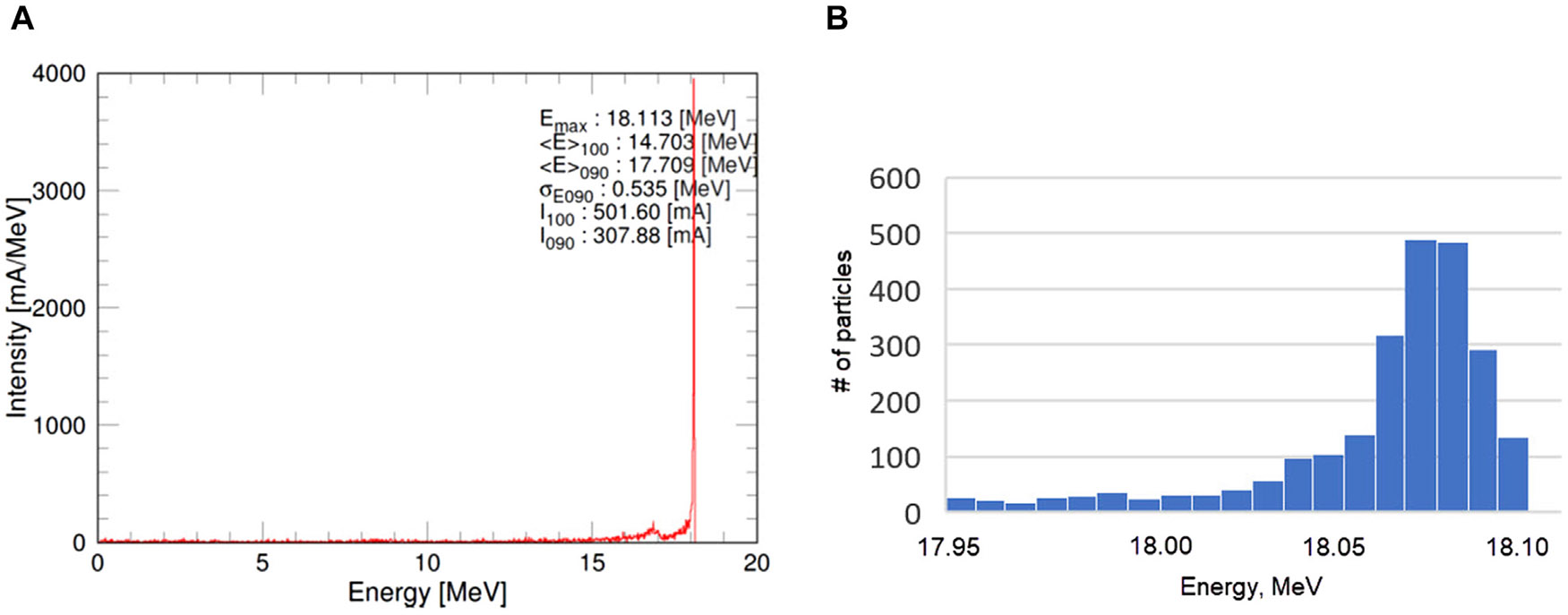
The energy spread of the beam accelerated to 18 MeV in wide range **(A)** and near the bunch head **(B)**. 10,000 particles were used for the simulation.

Electromagnetic simulations were performed in Superfish code, developed at Los Alamos National Laboratory (Young and Billen, 2003), which can accurately calculate 2D electric and magnetic field maps, which are then imported into GPT code (De Loos and Van Der Geer, 1996) for particle tracking simulations that can treat a space charge effect increasing the beam transmission ratio and avoiding large energy spread at the end of the accelerator. Figure 10 demonstrates the simulated energy spectrum of the accelerated beam, which quality is very good: narrow beam head and thin low-energy tail.

### 3.2 X-ray converter

**FIGURE 11:**
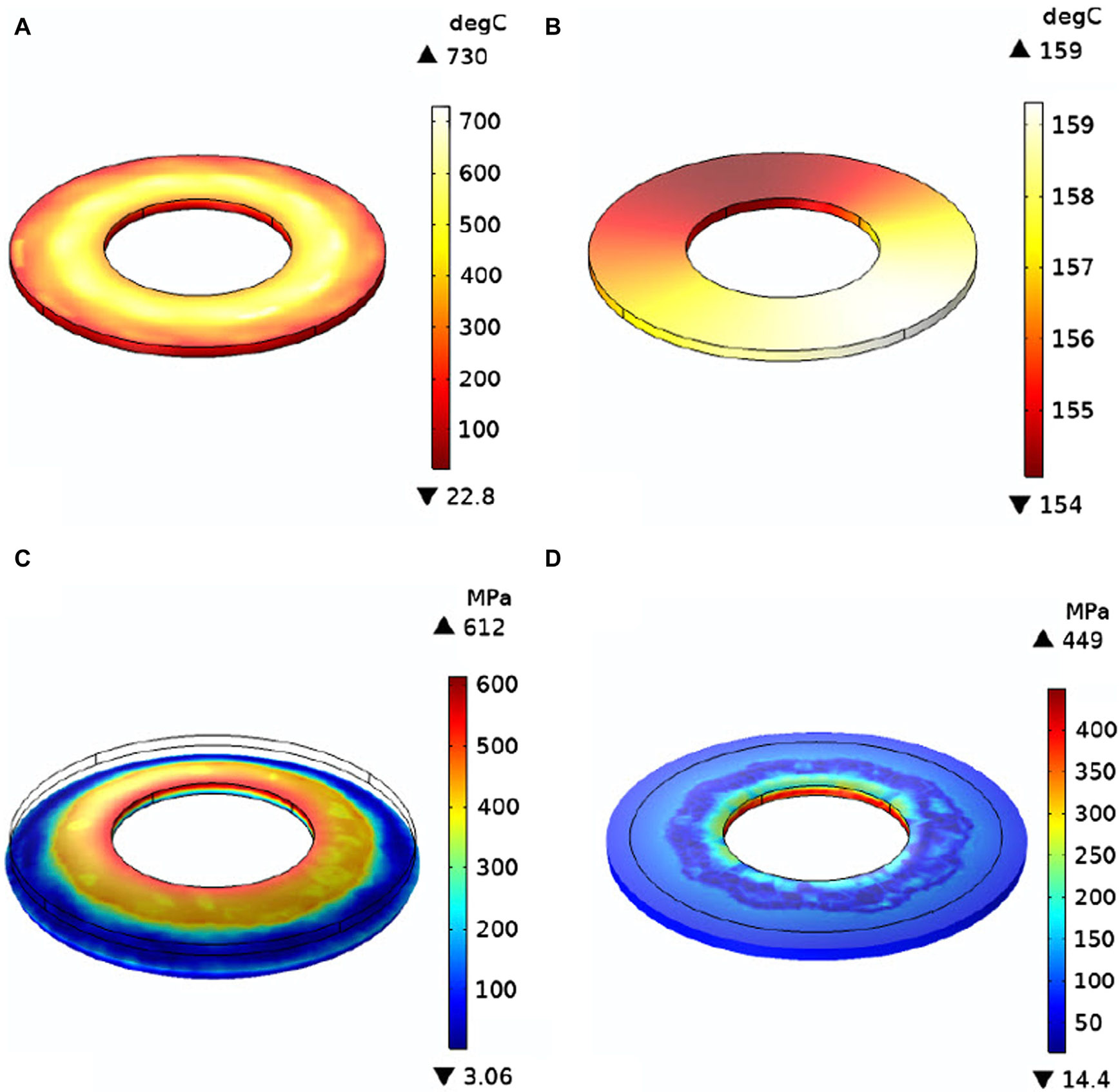
Simulated temperature **(A,B)** and mechanical stresses **(C,D)** maps after 1 s **(A,C)** and 10 s **(B,D)** of beam exposure.

After parametric study with various design parameters, a disk that satisfies these constraints has been designed. The key disk parameters are shown in Table 4. This target was modeled in COMSOL via finite-elements coupled with thermo-mechanical simulations to accurately verify its performance. Air cooling with a heat transfer coefficient of up to 100s W/m^2^·K after beam exposure. This film coefficient is achieved through a forced air convective effect produced by a combination of the high disk rotational velocity and externally fed air blade blowers. The plots in Figure 11 show the temperature and Von Mises stress distributions at different times after 1s of beam exposure. Elastic stresses result upon heating of the disk as a result of the temperature gradients in the disk, both radially and axially. The radial expansion of the disk against the constraint of inner surface rigid attachment manifest itself in in-plane stress distributions that generally image the temperature distributions. The main reason is that the local stress is dictated by the local temperature gradients, which are very steep around beam impact trajectories. Another source of stress and deformation is caused by axial temperature gradients, which produce bending-type deformations and axial stress distributions. Unlike conventional X-ray targets, it is important to recall that in FLASH radiotherapy, the treatment beam is only on for 1s, with a duty cycle defined by patient turnover periods, and therefore on the order of 10^−3^. With such a short beam exposure time, conventional conductive thermal management techniques, such as water cooling, which seek to establish thermal equilibrium at a steady state condition, are less effective in reducing the target temperatures due to the limitations of thermal diffusion.

These studies show that the X-ray target design that can sustain electron beams, capable of producing FLASH doses is feasible in terms of thermo-mechanical properties. The uniform heating is achieved when the rotational speed is as high as possible, with the limit being the centrifugal stresses, and with the speed being a noninteger multiple of the beam frequency. Future studies may explore the optimal strategy for beam heating in order to provide a stress pattern below the failure stress of W-5%Re, while obtaining the smallest possible disk for minimum material, engineering complexity and cost. The optimal target design parameters are summarized in Table 3.

## 4 Summary

The final parameters of the accelerator, simulated in Superfish and GPT are summarized in Table 4. These numbers demonstrate that the designed linac is capable of achieving FLASH doses within a room-size footprint by accelerating 325 mA beam to 18 MeV at 2.5% duty factor. The design became feasible thanks to the optimization of the energy parameters, L-band frequency choice, where commercial 10 MW klystron is available, design and optimization of the accelerator layout that allowed reducing the number of elements, bunching section design and application of solenoid. The expected dose meets the requirements for FLASH therapy but must be verified with Monte-Carlo simulations. Further dose improvement is possible by X-ray target optimization.

## 5 Discussion

Radiotherapy treats over 60% of cancer patients in the United States and is used in over 40% of curative cases. The success of radiotherapy depends on the therapeutic ratio, which is the efficacy of tumor killing over the degree of normal tissue damage. Ultra-high dose rate, aka FLASH, has recently attracted enormous research and clinical interest due to substantial therapeutic ratio gains across a wide range of cancers and normal tissue in mostly preclinical studies. FLASH is particularly appealing as it does not depend on exogenous agents such as radiosensitizers or protectants, thus bringing a potential mechanism for generalizable therapeutic ratio escalation. The major hurdle of clinical translation, however, is the availability FLASH capable radiation sources for human patients. Currently, protons are considered the readiest source for human FLASH therapy due to achievable instantaneous dose rate and radiological depth. However, other than the still limited availability of protons, there are technical challenges to delivering the entire dose with a FLASH dose rate without substantial compromise in physical dose conformality relying on scanning pencil beams and multiple angles. External beam X-rays produced via high-energy electrons hitting a target have been the most widely used modality for radiotherapy. The dose characteristics and intensity modulation of X-rays have been well understood and implemented. There are two major challenges to making X-ray FLASH available for human treatments: a mechanism for multidirectional beams and intensity modulation and an ultra-high dose rate linear accelerator. An important ongoing development in ultra-high dose rate medical linac is called PHASER (Maxim et al., 2019), which employs Distributed RF-coupling Architecture Genetically Optimized cell desigN (DRAGON), a plurality of distributed beamlines, and scanning electron beam on a stationary collimator for intensity modulation. PHASER is a remarkable combination of groundbreaking technologies that require substantial research and development. The approach we take is philosophically different from PHASER. Instead of developing completely new accelerator and intensity modulation technologies, we optimize existing technologies for FLASH X-rays. The rotational platform was previously published as Rotational direct Aperture optimization with a Decoupled ring-collimator (ROAD)^18^. We showed that achieving both the FLASH dose rate and superior dose conformity is possible. The current paper focuses on providing a technical roadmap for building an economical and practical high-output linear accelerator for ROAD X-ray FLASH delivery. The simulation study led to two connecting traveling wave guides achieving 18 MeV electron energy. The system maximally utilizes off-the-shelf components, including the klystron, modulator, and power supply. The system’s feasibility was simulated in detail to account for impedance, loss of efficiency due to thermal expansion, etc.

## 6 Conclusion

The paper describes a novel linear accelerator design capable of producing 100 Gy/s at 1 m for X-ray FLASH radiotherapy. Despite ~500x higher output than a standard clinical linac, the design utilizes available accelerator components for maximal practicality. The parameters of a rotating target capable of handling the high electron current are also determined.

## Figures and Tables

**TABLE 1:** Requirements to ROAD high-current electron linear accelerator for X-ray FLASH therapy, compared to the parameters.

RT type	FLASH	Conventional (Kutsaev et al., 2021c)	Conventional (Kutsaev et al., 2021b)
Frequency band	L	S	X
Energy [MeV]	18	9	6
Pulse length [us]	167	16	4
Rep rate [Hz]	150	250	250
Duty cycle [%]	2.5	0.4	0.1
Peak current [A]	0.325	0.13	0.125
Peak beam power [MW]	5.85	1.0	0.75
Dose rate* at 80 cm [Gy/s]	106.0	4.0	0.325

**TABLE 2:** RF parameters of the optimized CG TW accelerating structure.

Section	# of cells	Phase velocity/c	Aperture, mm	Q-factor	Shunt impedance, MΩ/m	Group velocity, %c
1	3	0.59	60.0	14,193	11.84	1.43
1	4	0.94	60.0	21,235	31.77	1.67
1	4	1.00	53.0	22,194	38.48	0.87
1	4	1.00	51.5	22,227	39.31	0.77
1	4	1.00	49.5	22,272	40.44	0.65
1	4	1.00	47.0	22,330	41.83	0.51
1	4	1.00	44.5	22,402	43.32	0.40
2	5	1.00	58.0	22,092	35.81	1.27
2	5	1.00	56.0	22,132	36.86	1.10
2	4	1.00	54.0	22,173	37.94	0.94
2	4	1.00	52.0	22,217	39.04	0.80
2	4	1.00	49.5	22,272	40.44	0.65
2	4	1.00	47.0	22,330	41.83	0.51

**TABLE 3:** Optimal target design parameters.

Parameter units	Value
Rotation speed	17,123 RPM
Inner diameter	5 cm
Outer diameter	10 cm
Thickness	3 mm
Weight	0.5684 Kg
Adiabatic temperature	3136.2°C
Tangential stress	535.6 MPa
Safety Factor	1.4

**TABLE 4:** Beam parameters of the designed linac.

Injector	30 kV DC gun, 600 mA
Accelerating section	2 x 2 m-long TW CG sections
Beam current (peak)	325 mA
Beam current (average)	8.1 mA
Beam energy (max)	18.07 MeV
Beam energy spread	10 keV
Expected dose @80 cm (analytical)	105 ± 10 Gy/s

## Data Availability

The raw data supporting the conclusion of this article will be made available by the authors, without undue reservation.
